# Sub-Sampling Framework Comparison for Low-Power Data Gathering: A Comparative Analysis

**DOI:** 10.3390/s150305058

**Published:** 2015-03-02

**Authors:** Bojan Milosevic, Carlo Caione, Elisabetta Farella, Davide Brunelli, Luca Benini

**Affiliations:** 1 DEI, University of Bologna, 40123 Bologna, Italy; E-Mails: carlo.caione@unibo.it (C.C.); elisabetta.farella@unibo.it (E.F.); luca.benini@unibo.it (L.B.); 2 E3DA, Fondazione Bruno Kessler, I-38123 Trento, Italy; 3 DII, University of Trento, I-38123 Trento, Italy; E-Mail: davide.brunelli@unitn.it; 4 IIS, ETH, 8092 Zurich, Switzerland

**Keywords:** wireless sensor networks, low-power design, data reconstruction, compressive sensing, latent variables

## Abstract

A key design challenge for successful wireless sensor network (WSN) deployment is a good balance between the collected data resolution and the overall energy consumption. In this paper, we present a WSN solution developed to efficiently satisfy the requirements for long-term monitoring of a historical building. The hardware of the sensor nodes and the network deployment are described and used to collect the data. To improve the network's energy efficiency, we developed and compared two approaches, sharing similar sub-sampling strategies and data reconstruction assumptions: one is based on compressive sensing (CS) and the second is a custom data-driven latent variable-based statistical model (LV). Both approaches take advantage of the multivariate nature of the data collected by a heterogeneous sensor network and reduce the sampling frequency at sub-Nyquist levels. Our comparative analysis highlights the advantages and limitations: signal reconstruction performance is assessed jointly with network-level energy reduction. The performed experiments include detailed performance and energy measurements on the deployed network and explore how the different parameters can affect the overall data accuracy and the energy consumption. The results show how the CS approach achieves better reconstruction accuracy and overall efficiency, with the exception of cases with really aggressive sub-sampling policies.

## 1. Introduction

The recent technological evolution of sensing devices for wireless sensor networks (WSNs) has triggered research activities in the field of data gathering and compression. The goal is to minimize sampling, storage and communication costs to extend as much as possible the lifetime of the network, while maintaining the desired data accuracy [1].

In a typical WSN scenario, the communication pattern consists of a very large number of small and low-cost devices that periodically sample the sensors' data and transmit them to a central collecting sink [2]. For large scenarios, where the device transmission range is smaller than the area to cover, multi-hop communication is used to efficiently gather the desired data. In this case, the network topology organization and the data transmission and routing approaches should be optimized to ensure the correct network coverage and connectivity and to balance data acquisition quality and power consumption [3].

Common approaches to extend the uptime of a sensor node try to enhance the battery life directly by harvesting energy from the environment and employing low-power hardware architectures [4] or using improved wireless protocols and distributed data processing [5]. More recently, researchers have been optimizing the battery life indirectly by reducing the overall amount of data sensed and transmitted through the network [6,7]. When the considered signals present spatio-temporal correlations, those can be used to reconstruct the desired data from only a limited portion of collected samples [8]. The ability to reconstruct missing data enables the adoption of aggressive duty cycling policies on individual sensor nodes, sampling only a limited part of the data, thereby reducing the energy consumption [9].

Among the different proposed approaches to increase the energy efficiency of a sensor network, we focus on strategies that leverage correlations to reconstruct sub-sampled signals. Two of the most promising techniques capable of successfully recovering the signal from highly incomplete versions are compressive sensing (CS) [10,11] and a data-driven statistical model based on latent variables (LV) [12,13]. These techniques are both able to recover the original signals from a smaller sub-sampled version obtained by skipping samples during the acquisition phase.

CS theory claims that if a signal can be compressed using classical transform coding techniques and its representation is sparse on some basis, then a small number of projections on random vectors contains enough information for approximate reconstruction [10]. Natural signals have usually a relatively low information content as measured by the sparsity of their spectrum [14]; therefore, the theory of CS suggests that randomized low-rate sampling may provide an efficient alternative to high-rate uniform sampling. This peculiar form of CS is a novel strategy to sample and process sparse signals at a sub-Nyquist rate [15].

The second considered framework is an energy-efficient and data-driven technique to estimate missing data within a heterogeneous sensor network, based on latent variables [12]. This approach extends the standard latent variable factorization model [16], which typically considers only dyadic interactions in data, to multivariate spatio-temporal data, by applying tensor decomposition techniques [17]. The key advantage of using a latent variable model is that it provides a compact representation of the gathered data that can be used to recover the missing samples. To perform well under extreme sub-sampling conditions, the standard technique is extended to explicitly incorporate the spatial, temporal and inter-sensor correlations present in the considered data [13].

In this work, we focus on the comparison between these two approaches and their impact on the energy consumption of the network nodes. We describe a real deployment of a WSN for the environmental monitoring of a heritage building, which is used as a common platform and testbed for the evaluation. The two techniques share the same approach, but address the reconstruction problem from different theoretical bases; hence, a straightforward question is which one is most well suited as an energy-minimization technique for WSNs. To the best of our knowledge, this is the first work in which the two techniques are directly compared, using the same dataset and a detailed power consumption model to evaluate their reconstruction abilities and the energy efficiency.

To compare the performance of the two techniques, evaluating the best trade-off between signal recovery and energy savings, we explore the impact of features, such as the use of network-wide correlations to improve the reconstruction accuracy or the length of the data block to be reconstructed. For both the hardware characteristics and the gathered data, we use a real deployed sensor network. The power consumption measurements are used to model the node's power consumptions to allow an easy evaluation of the energy expenditure for the different algorithms and sampling strategies used.

The rest of the paper is organized as follows: In Section 2, the related works are introduced. In Sections 3 and 4, the mathematical background for CS and LV is presented, while in Section 5 the models for the hardware, network and the power consumption are described. The simulation results are shown in Section 6, and the conclusions are in Section 7.

## 2. Related Work

The problem of data gathering, compression and signal reconstruction in WSNs is well explored in the literature. Even though the majority of the works deal with reconstruction algorithms and mathematical aspects, practical aspects and low power implementation problems have lately been gaining interest.

### 2.1. Theoretical Approaches

As seen in the previous section, CS builds on several works, like [10,11], which show that if a signal can be compressed using classical transform coding techniques and its representation is sparse on some basis, then a small number of projections on random vectors contain enough information for approximate reconstruction.

For WSN data gathering applications, CS can be used to improve the overall efficiency of the network. One approach is to use CS to aggregate the data as it is collected by a multi-hop network, avoiding a progressive increase of data to be transmitted as we travel from the peripheral nodes to the sink. The work in [18] introduces an approach to minimize the network energy consumption through joint routing and compressed aggregation. The approach has an optimal solution, which is NP-complete, so the authors present a greedy heuristic that delivers near-optimal solutions for large-scale WSNs. Another data acquisition protocol for WSNs is presented in [19], where the results from studies in vehicle routing are applied to data collection in WSN and integrated with CS. Furthermore, this approach is NP-hard, and the authors propose both a centralized and a distributed heuristic to reduce computational times and to improve the scalability of the approach. These works analyze the routing and aggregation problem in WSNs, while in our work, we focus on the spatio-temporal correlation in the observed phenomena, which can be used to improve the data reconstruction accuracy Our approach allows the sensor nodes to duty-cycle and skip a significant amount of samples, thus saving energy Our analysis focuses on joint data reconstruction and on power consumption impacts in a real deployment scenario.

When CS is used in a scenario where several sensors acquire data from the same environment, we can think that the sensed data have a certain kind of shared information that can be exploited to perform a better reconstruction. The best known technique used for exploiting the inter- and intra-correlation among several nodes in a WSN is the distributed CS (DCS) introduced in [20]. In these works, the authors analyze three different Joint Sparsity Models (JSMs): JSM-1, JSM-2 and JSM-3, to describe most of the signal ensemble; and for each sparsity model, they present a different reconstruction algorithm. Among other works dealing with joint sparse recovery, we can cite [21] or Kronecker CS, introduced in [22]. Different from these works, in this paper, we want to focus on a special technique for the reconstruction of jointly sparse solutions, known as multiple measurement vector (MMV), based on *ℓ*_2,1_-regularization, which is introduced in [23].

Latent variables and decomposition-based techniques have also been proposed in the literature, with their most notable applications in collaborative filtering [24] and recommender systems [25]. A standard way to learn a set of latent variables for a two-dimensional dataset is to apply a matrix factorization technique. A recent review of matrix factorization techniques with applications to recommender systems can be found in [16]. To achieve a better latent variable model and to allow the prediction of the missing entries in the original data matrix, standard matrix factorization techniques have been extended to incorporate temporal dynamics and, thus, better capture the temporal evolution of the data [12,26]. When dealing with multivariate datasets, which can be represented by *N*-dimensional data arrays, tensor factorization techniques can be exploited to further improve the performance of the models [27]. In our work, we applied a tensor factorization approach to WSNs, taking advantage of the three-dimensional nature of the data gathered from a heterogeneous sensor network. We further extended this approach, incorporating for each data dimension a correlation model learned from the data itself, to enhance the capabilities to reconstruct the missing data.

### 2.2. WSN-Related Practical Implementations

The general problem of using CS in WSNs is investigated in several works. In [28], the measurement matrix is created jointly with data routing policies, trying to achieve an efficient reconstruction quality. Furthermore, in [29], the authors try to improve the reconstruction by reordering the input data to achieve a better compressibility. The main focus of these works is to investigate the signal reconstruction problem, but they are missing the consideration about how the usage of CS impacts the power consumption. While there is no doubt that CS is a powerful technique for data size reduction and compression, its usage and impact on network lifetime, when real hardware and COTSnodes are used, is still marginally addressed in literature.

The work in [30] is one of the first papers trying to address the issue of energy consumption for data compression, dealing with the problem of generating a good measurement matrix using as low energy as possible. In this work, the research is focused on wireless body sensor networks (WBSNs) for real-time energy-efficient ECG compression. This is quite a different application when compared to WSNs, where the presence of several nodes, sensing the same environment, permits one to exploit the distributed nature of the signals to improve the quality of the data recovery.

Other works deal with the use of CS when data are gathered from different joint sources, using DCS to improve the recovery quality. In [31], DCS and principal component analysis are used to reconstruct spatially- and temporally-correlated signals in a sensor network, but once again, the contribution of the power consumption for compression in the network lifetime is neglected.

In this paper, we deal with CS when the signals are sampled at a sub-Nyquist frequency, resembling a technique that is referred to analog CS in the literature. The name derives from the fact that the sub-sampling is performed by dropping samples during the acquisition and analog-to-digital conversion (ADC) stage [32]. One notable example of this technique is in [33], where the effects of circuit imperfections in the analog compressive sensing architectures are discussed. In the framework proposed in this work, samples are not discarded by analogue circuits, but are not sampled at all, saving the energy for waking up the node.

In the literature, other works investigate the problem when the samples are discarded by the device performing the sensing rather than the ADC. For example, in [34,35], the analysis of energy consumption is totally neglected, and the recovery is strictly related to the specific applications described. Different from environmental signals, used in our work, the signals coming from the oximeter present a much higher temporal correlation, with small variations in their temporal evolution, facilitating their reconstruction.

Matrix factorization learning of latent variables has been used for recovering missing data in sensor networks in [12], where temporal correlations found in the dataset are used to infer the missing variables. Tensor decomposition techniques have been applied on WSNs in [36], where the learned models are used to find the damage in a structural health monitoring application. The previously presented algorithms only consider homogeneous sensor streams, dealing with one sensor at a time, and do not consider the energy costs across the network. Instead, our approach focuses on the multivariate nature of the collected data, and it expands the tensor factorization techniques by employing spatio-temporal and intra-sensor correlations for more robust and better results than the existing methods.

The energy consumption during sub-sampling operations is instead considered in [37], where the authors use a sparse generated matrix adjusting the sampling rate to maintain an acceptable reconstruction performance, while minimizing the energy consumption. Different from our work, they do not consider inter- and intra-correlation among signals and do not use any group sparsity-enhancing algorithm to perform a better recovery. In addition, our work presents a real network deployment and takes advantage of the gathered data and power consumption measurements to accurately evaluate the two proposed frameworks in a common testbed.

## 3. Compressing Sensing and Group Sparsity

### 3.1. Mathematical Background

Considered a continuous signal *x*(*t*) of duration *T*, and *x*(*n*) 1 ≤ *n* ≤ *N* is its discrete version. The Nyquist theorem states that in order to perfectly capture the information of the signal *x*(*t*), having a bandwidth of *B*_nyq_/2 Hz, we must sample the signal at its Nyquist rate of *B*_nyq_ samples per second.

In formula:

(1) $$x\left(n\right)={x\left(t\right)|}_{t=nT_{s}}$$

where *T_s_* ≤ 1/*B*_nyq_ and *NT_S_* < *T*. The sampled signal *x*(*n*) is represented by an *N*-dimensional vector of real numbers **x**.

If the vector **x** is sparse, then CS is able to recover this finite-dimensional vector **x** ∈ ℝ*^N^* from a very limited number of measurements of the original signal *x*(*t*). The sparsity of a signal ***α*** is usually indicated as the ℓ_0_-norm of the signal, where the ℓ*_p_*-norm ‖ · ‖*_p_* is defined as:

(2) $${\left\|\alpha \right\|}_{p}={\left(\sum_{i=0}^{N-1}{\left|\alpha_{i}\right|}^{p}\right)}^{1/p}$$

with ***α*** ∈ ℝ*^N^*. If the signal **x** is sparse, then there exists some *N* × *N* basis or dictionary **Ψ** ∈ ℝ*^N^*^×^*^N^*, such that there is an *N*-dimensional vector ***α*** implying **x** = **Ψ*α*** and ‖***α***‖_0_ ≤ *K* with *K* ≪ *N*. CS theory demonstrates that it is possible to compress this kind of sparse signal using a second different measurement matrix **Φ** ∈ ℝ*^M^*^×^*^N^* with *M* ≪ *N* [11]. The compression can be written as **y** = **Φx**, where **y** is the *M*-dimensional measurements vector. While **Ψ** is usually defined by the signals characteristics, **Φ** has to be designed so that *M* is much smaller than *N*.

Having the measurements vector **y**, the recovery of the original signal **x** can be obtained by the inverse of the measurement problem:

(3) y=Θα=ΦΨα

Even though the inversion is not an easy task, since the matrix **Θ** ∈ ℝ*^M^*^×^*^N^* is rectangular with *M* ≪ *N*, the fact that **x** is sparse can relax the problem by opening the way to the use of optimization-based reconstruction or iterative support-guessing reconstruction.

The most common optimization-based method, here reported for the sake of clarity, is the basis pursuit (BP) [38] method that searches for the most sparse solution for which the ‖***α***‖_1_ is minimum:

(4) $$\hat{\alpha }=\arg\min\;{\left\|\alpha \right\|}_{1}\;\text{subject to}\;y=\Theta \alpha =\Phi \Psi \alpha$$

CS theory proves that if the two matrices **Φ** and **Ψ** are incoherent (elements of the matrix **Φ** are not sparsely represented in the basis **Ψ**) and the original signal x is compressible or sparse, we can recover ***α*** with high probability.

To further enhance the recoverability, recent studies propose taking into account additional information about the underlying structure of the solutions [39]. When the signals to compress and recover are obtained from sensors deployed close to each other in the environment, we can expect that the ensemble of these signals presents an underlying joint structure. This characteristic can be exploited to further compress the data, without a loss in reconstruction accuracy In practice, this class of solutions is known to have a certain group sparsity structure. This means that the solution has a natural grouping of its components, and the components within a group are likely to be either all zeros or all non-zeros. Encoding the group sparsity structure can reduce the degrees of freedom in the solution, thereby leading to better recovery performance.

Having an ensemble of *J* signals, we can denote each signal with **x***_j_* ∈ ℝ*^N^* with *j* ∈ {1,2,…, *J*}. For each signal **x***_j_* in the ensemble, we have a sparsifying basis **Ψ** ∈ ℝ*^N^*^×^*^N^* and a measurement matrix **Φ***_j_* ∈ ℝ*^M^*^×^*^N^*, such that, as before, **y***_j_* = **Φ***_j_***x***_j_* with *M_j_* ≪ *N* and **x***_j_* = **Ψ*α****_j_*. The reconstruction of jointly sparse solutions, also known as the multiple measurement vector (MMV) problem, has its origin in sensor array signal processing and recently has received much interest as an extension of the single sparse solution recovery in compressive sensing. The recovery problem can be formulated as:

(5) $$\begin{array}{cc} \underset{\tilde{\alpha }}{\min} & {\left\|\tilde{\alpha }\right\|}_{w,2,1}:=\overset{n}{\underset{i=1}{\text{∑}}}w_{i}{\left\|{\tilde{\alpha }}_{i}\right\|}_{2}\\ \text{subject to} & \tilde{\Theta }\tilde{\alpha }=\tilde{\text{Y}} \end{array}$$

where $\tilde{\text{Y}}={\left[{\text{y}}_{1}^{T}{\text{y}}_{2}^{T}\ldots {\text{y}}_{J}^{T}\right]}^{T}$, $\tilde{\alpha }={\left[\alpha_{1}^{T}\alpha_{2}^{T}\ldots \alpha_{J}^{T}\right]}^{T}$, *w_i_* is the weight and ***Θ̃*** ∈ ℝ*^JM^*^×^*^JN^* is a matrix having on the diagonal matrices **Θ***_j_* = **Φ***_j_***Ψ** for *j* ∈ {1,2,…, *J*}.

### 3.2. CS and Sub-Nyquist Sampling

As discussed in the previous section, to successfully recover the original signal from its sampled version, the samples are taken regularly on a time axis at a given rate that is not less than the Nyquist one. With respect to CS, this requirement means that the measurement matrix **Φ** is a dense matrix (usually an independent and identically distributed Gaussian matrix). A particular form of CS, usually referred to as analog CS, relies on random sampling and aims to produce a number of measurements fewer than with the Nyquist sampling, still enabling the reconstruction of the original signal. While analog CS is usually performed by means of specialized hardware encoders, this is also a suitable technique to be performed on WSNs nodes, opportunely skipping samples during the acquisition phase.

From a mathematical point of view, the problem is still the same as the problem in Equation (3); what is different is the form of the measurement matrix **Φ**, which is not a dense matrix, but is a sparse measurement matrix. More precisely, if **B** is an *M*-dimensional vector where each element is a unique random entry between one and *N*, then the matrix **Φ** in the analog CS is a sparse *M* × *N* measurement matrix, which is composed by an all-zero vector on each row and a “1” at the location given by the *i*-th element of **B**. This is a very simple measurement matrix, energetically inexpensive to generate and store and permits a huge reduction in the duty-cycling of the nodes.

## 4. Latent Variables and Tensor Factorization

Latent variable-based factorization is a simple, yet powerful framework for modeling data and has been successfully applied in several application domains [16]. The main idea behind this framework is to model the large number of observed variables (the observed data) in terms of a much smaller number of unobserved variables (the latent variables). The latent variables are learned from the observed data and are used to estimate the missing samples, modeling complex interactions between the observed variables through simple interactions between the latent variables.

More specifically, given some multivariate data that are collected by a heterogeneous WSN in a large field over time, we can naturally organize these into a three-dimensional data array (or a three-tensor). Each of the three dimensions corresponds to a different variate of a particular measurement (e.g., the time, the location and the sensor type associated with each reading). Once the data are organized in this way, we can then associate a low-dimensional latent variable with each unique location, time slice and sensor type. We can thus model a particular observation (at a given location, time and type) as a noisy combination of the associated latent variables. In many scenarios, a multiplicative combination of these latent variables is able to capture intricate dependencies in the data [17,40]. The goal then is to learn a good set of latent variables (that is, find a factorization) that can efficiently represent our observed data.

### 4.1. Modeling Details

We now formally define our model. For each unique time instance *t*, sensor type *s* and node location *n*, we associate a unique *K*-dimensional vector, *a_t_*, *b_s_* and *c_n_*, respectively. These unobserved vectors are called the latent factors or variables and are assumed to control the location-, time- and sensor-specific interactions present in the observed data.

**Figure 1.**
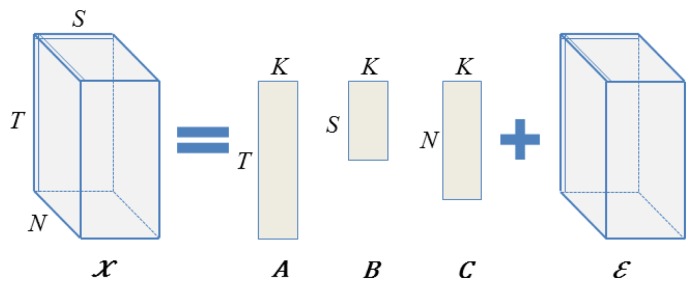
Tensor data representation and its matrix factorization.

Then, given a [*T* × *S* × *N*] tensor *χ* of sensor readings from *S* different sensor types collected at *N* different nodes and *T* different time instances, with possible missing entries, we model *χ* as follows (see Figure 1). We assume that each reading *x_tsn_* (the reading at time *t*, for sensor type *s*, at node location *n*) is a noisy realization of the underlying true reading that is obtained by the interaction of the time-specific latent variable *a_t_*, with the sensor-specific latent variable *b_s_* and with the location-specific variable *c_n_*.

That is,

(6) $$x_{\text{tsn}}=\sum_{k=1}^{K}a_{tk}b_{sk}c_{nk}+\varepsilon$$

where *ε* is modeled as independent zero-mean Gaussian noise (*ε* ∼ 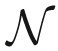
(0, *σ*^2^)). Observe that under this model, once all of the latent variables are known, one can recover the true readings of all sensors at all locations and times. Thus, the goal is to find the most predictive set of vectors *a_t_*, *b_s_* and *c_n_* for all *t* = 1,…, *T, s* = 1,…, *S*, *n* = 1,…, *N*. Such a representation models the entire data of size [*T* · *N · S*] by just [*K* · (*T* + *N* + *S*)] modeling parameters. The choice of the free parameter *K* provides a key trade-off: a large *K* increases the number of modeling parameters and, thus, can help model the observed data exactly. However, this lacks the capability to predict unobserved/missing data due to overfitting. A small *K*, on the other hand, escapes the overfitting problem, but the corresponding model lacks sufficient richness to capture salient data trends. The exact choice of a good *K* is typically application dependent and is derived empirically.

#### 4.1.1. Learning the Latent Variables

Finding the optimal set of *K*-dimensional latent variables given the observations is equivalent to factorizing the given tensor into three matrices, each of rank at most *K* [17]. Thus, assuming that all of the data is known (that is, every entry in the tensor is observed), we can find the latent factors by employing the Canonical decomposition/Parallel factors (CP) tensor factorization [17]. This is simply a higher-order generalization of the matrix singular value decomposition (SVD) and decomposes a generic third order tensor in three matrix factors *A*, *B* and *C*. Restricting the ranks of each of the matrix factors to at most *K* yields the best rank *K* approximation. Algorithmically, the matrix factors are found by an alternating least squares approach (ALS) [40], which starts from a random initialization and iteratively optimizes one matrix factor at a time, while keeping the other two fixed.

This technique can be generalized to work with tensors that have missing entries. Since sensor nodes can periodically go offline due to duty-cycling or running out of energy (preventing all sensors on a node from collecting any data for an extended period of time), we need to extend our basic model to deal with data missing from multiple sensors or nodes, resulting in entries and rows of missing data in the collected tensor. In order to do well in this regime, we extend the basic tensor factorization model to explicitly incorporate spatially-, temporally- and sensor-specific information from neighboring observations by explicitly learning and enforcing the corresponding correlations.

### 4.2. Incorporating Correlations

To successfully interpolate the sensor interactions to contiguously missing blocks of data, we need to explicitly model spatially-, temporally- and sensor-specific trends within each of our latent variables *a_t_*, *b_s_* and *c_n_*. Such trends ensure that the latent variables *a_t_* and *a_t′_* (respectively, *b_s_* and *b_s′_* and *c_n_* and *c_n_*_′_) take similar values when times *t* and *t′* are “similar” (respectively, sensors types *s* and *s′* and locations *n* and *n′*). Note that similarity can mean anything based on the context. For locations, it can mean that variables associated with two locations that are close in distance should have similar characteristics, while for time, it can mean that variables associated with times that are the same hour of the day or the same day of the week should have similar characteristics. Here, we take a data-driven approach to infer the best notion of similarity using correlations directly computed from the data. We use a limited sample of data collected from the network to learn the correlations, which are then applied to the subsequent slot of data to reconstruct the unsampled values.

The similarity constraints are modeled in the same way for all three sets of latent variables, and here, we illustrate the case for the *a_t_*'s. Since each *a_t_* is a *K*-dimensional variable, let *a_tk_* denote its *k* – th coordinate. We model *a_tk_* (independently for each coordinate *k*) as:

(7) $$\begin{array}{cc} a_{:k} & =\mu_{ak}+\alpha_{:k}\\ \alpha_{:k} & ∼N\;\left(0,\Sigma_{a}\right) \end{array}$$

Here, *a*_:_*_k_* represents the collection of all *a_t_'*s (across *t* = 1,…,*T*) in the *k*–th coordinate and μ*_a_* represents their mean value. The distributional constraint over *α*_:_*_k_* (as 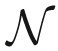
(0,Σ*_a_*)) enforces the similarity constraints via the *T* × *T* covariance matrix Σ*_a_*. By changing the *t*, *t*′ entry of Σ*_a_*, we can encourage/discourage the corresponding *a_t_* and *a_t′_* to take similar values: a high positive value at Σ*_a_*(*t*, *t*′) encourages a positive correlation; a high negative value encourages a negative correlation; while a value close to zero does not encourage any correlation. We enforce similar constraints on *b_s_* and *c_n_* variables, as well.

To get the right similarity constraints Σ*_a_*, Σ*_b_* and Σ*_c_* (for latent variables *a_t_*, *b_t_* and *c_n_*), we compute them from the data that we are considering. For spatial similarity constrains, we computed the averaged pairwise Pearson correlation coefficients between data from different pairs of locations and averaged over different times and sensors. We do the same to approximate inter-sensor and temporal similarities. We can have only one global correlation constraint along each of the dimensions.

### 4.3. Parameter Learning

We can learn the underlying latent variables in a probabilistic framework using a maximum *a posteriori* (MAP) estimate. In particular, let θ denote all of the model parameters (*i.e.*, θ = {{*a_t_*}, {*b_s_*}, {*c_n_*}, σ}), then the optimum choice of parameters θ*_MAP_* given the data *χ* is obtained by:

$$\begin{array}{cc} \theta_{\text{MAP}}\left(\chi \right):=\underset{\theta }{\arg\max}\underset{\text{likelihood}}{\underset{︸}{p\left(\chi \;|\;\theta \right)}}\underset{\text{prior}}{\underset{︸}{p\;\left(\theta \right)}} & =\underset{\theta }{\arg\max}\underset{t,s,n\in \text{observed}}{\text{∑}}\log p\left(x_{\text{tsn}}\;|\;a_{t},b_{s},c_{n},\sigma \right)+\\ & +\overset{K}{\underset{k=1}{\text{∑}}}\log p\left(a_{:k}\right)+\overset{K}{\underset{k=1}{\text{∑}}}\log p\left(b_{:k}\right)+\overset{K}{\underset{k=1}{\text{∑}}}\log p\left(c_{:k}\right). \end{array}$$

The first term (the likelihood) takes the form of Equation (6), and the other terms represent the priors for each latent variable; each one of them takes the form of Equation (7). We take a uniform prior over σ, the standard deviation of the residuals in Equation (6), so it does not explicitly show in the equation.

This optimization does not have a closed form solution. Standard gradient-based techniques can be used to get a locally optimal solution. Here, we can follow an alternating hill-climb approach by optimizing the value of one variable while keeping all others fixed to get a good solution. As for the standard factorization technique, this iterative approach uses a random initialization of the latent variables.

## 5. Hardware, Network and Power Models

In this section, we will describe the hardware and software characteristics of the WSN deployed as a testbed for this work [41]. In particular, we report the experience gained during the EU Project 3ENCULT (Efficient energy for EU cultural heritage, http://www.3encult.eu) regarding the design and implementation of a WSN for environmental monitoring of heritage buildings. We present the network solution developed to efficiently satisfy the requirements for long-term monitoring of a historical building, called Palazzina della Viola, located in the center of Bologna, Italy.

### 5.1. Hardware

**Figure 2.**
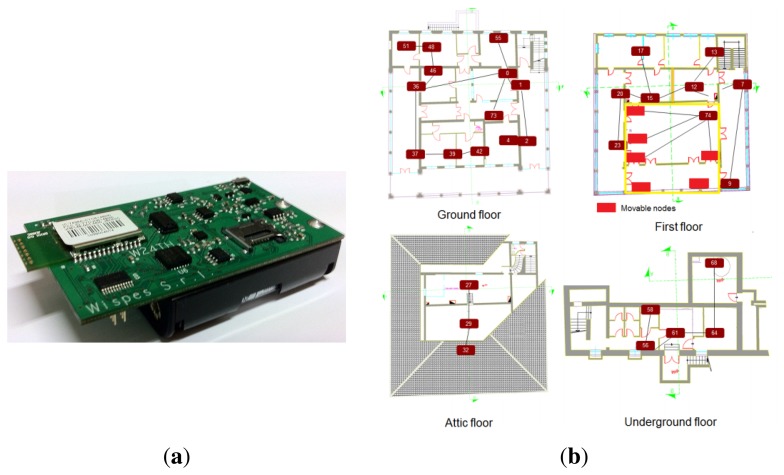
**(a)** The W24TH sensor node and **(b)** the deployment map.

The sensor nodes used in the network are the W24TH nodes, shown in Figure 2a. The hardware core of each node is based on the NXP JN5148 module, which is an ultra-low-power, high-performance wireless microcontroller targeted at ZigBee PRO networking applications. The device features an enhanced 32-bit RISC processor, a 2.4-GHz IEEE 802.15.4 compliant transceiver, 128 kB of ROM, 128 kB of RAM and several analogue and digital peripherals. Its power consumption is 15 mA for transmission and 18 mA for reception, which gives 35% of power saving when compared to other similar platforms (e.g., TelosB).

The board is equipped with several sensors such as a temperature, humidity, ambient light and a metal-oxide gas sensor. Each sensor is equipped with a specific conditioning circuit, which is useful for various ambient monitoring applications. A MicroSD card reader is also available, intended for local back-up, data logging and firmware updates. Moreover, each node is equipped with a USB battery charger, a 32-kHz quartz oscillator and an expansion connector. The power management subsystem provides the ability to switch off the whole system by software, thus enabling a 10 μA sleep mode current consumption. In addition, all sensors can be individually disconnected for the efficient management of the hardware resources, reducing the node's power consumption.

For our installation, a network of 23 low-power sensor nodes is deployed across the three floors of the historic building, as shown in Figure 2b. The topology is a tree network with a central collecting sink that is in charge of gathering the data from the nodes and forwarding them to a remote PC, used to log the data and reconstruct the missing samples.

### 5.2. Network

Generally, in low-power sensor nodes, the communication subsystem has a much higher energy consumption than the computation and sensing subsystems; hence, the radio management and duty cycling are crucial aspects for energy efficiency. Ideally, we could turn on the radio as soon as a new data packet becomes available for sending and switch it off again when the data are sent. The problem with this technique is that in collaborative networks, such as WSNs, coordination among nodes is required to enable data exchanging and forwarding from the source node to the sink. Thus, a sleep/wakeup scheduling algorithm is required to permit the correct operation of the network.

To solve this problem, we developed and implemented a new power management and scheduling protocol, named conservative power scheduling (CPS). This protocol is built on top of the IEEE 802.15.4 MAC protocol and adapts a slotted approach derived from time division multiple access (TDMA) schemes for the staggered wakeup pattern [42].

CPS is based on a centralized approach and provides a time synchronization by using a non-beacon-enabled mode and the dissemination of a service-packet. Moreover, the algorithm avoids collisions during the data packet transmission through a TDMA technique, where each node in the network transmits its data in a given time slot. The service-packet contains several pieces of information, including data for time synchronization, as well as the sequence of active sensors for all receivers. The transmission of the service-packet across the network is performed by using a novel approach based on a constructive interference of IEEE 802.15.4 symbols for fast network flooding and implicit time synchronization [43].

In this protocol, each communication period, identified by a service-packet, is divided into two parts: an active interval (AI), during which the node must keep its radio on to exchange packets with other nodes, and a sleep interval (SI), during which the node is sleeping. To permit the correct data exchange through the network, the talk interval between a node and its parent/child must overlap for at least a time slot, needed to exchange the desired data.

The real challenge of the CPS protocol is to correctly assign the time slots to each node, so as to minimize the power consumption. To achieve this goal, each node has to be in the active interval for the shortest time possible, waking up just in time for sending sampled data to the parent node and going back to sleep as soon as it has forwarded all of the data coming from its child nodes. Different from other approaches found in the literature [44], CPS does not calculate at run-time the slot scheduling, but it uses a static scheduler computed off-line. The algorithm for slot assignment takes as input the tree-structure of the network and gives as output the slot times assigned to each node.

**Figure 3.**
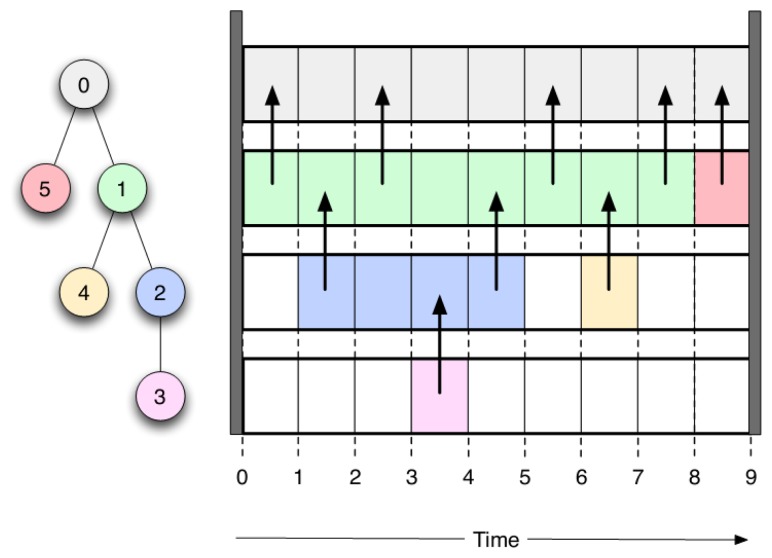
Example of scheduling using the conservative power scheduling (CPS) protocol.

The algorithm is articulated in two different steps: (1) ordering and (2) scheduling. In the ordering phase, each node in the network is assigned a unique ID that defines the priority of the node, following a depth-first approach. After each node is opportunely tagged with an ID, the scheduling step of the algorithm is in charge of computing the start and duration of the active time slot for each node. It ensures that each node waits until data sent by its own parent have reached the sink before sending out its own data, avoiding collisions in the network. Afterwards, the node can switch to sleep mode only after having forwarded data coming from all of its child nodes. An example of the protocol's scheduling is illustrated in Figure 3.

In a practical implementation of CPS, the synchronization using broadcast packets from the coordinator can be problematic due to the delay in the packet relay through the network. For this reason, the margin for synchronization is relaxed, accounting for clock drift and delays in the transmission of synchronization packets.

### 5.3. Power Model

We introduce an architecture-level power model to evaluate the energy consumption of the node when the sub-sampling parameters are changed. Using this power model, fitted and validated with data from real hardware and measurements, we can easily evaluate how changing the parameters influences the energy consumption and the lifetime of the network.

Starting from the assumption reported in the previous section, the average energy consumption in each period of duration *T_k_*, for a sub-sampling factor *ρ* applied to a block of *N* sampling intervals, is:

(8) $$E_{k}=\rho \;\left(E_{\text{setup}}+E_{\text{sampl}}+E_{\text{store}}\right)+E_{\text{sleep}}+N^{-1}\;\left(E_{\text{nv}}+E_{\text{send}}\right)$$

where *E*_sleep_ is the energy spent in sleep mode, *E*_setup_ is the energy used for waking up and setting up the device, *E*_Sample_ is the energy for sampling each sensors, *E*_send_ is the energy used to send the acquired data, *E*_store_ is the energy to store the acquired sample in non-volatile memory and *E*_nv_ is the energy spent to read the data from non-volatile memory.

Expanding each term, we have:

(9) $$E_{k}=\rho \left(T_{\text{setup}}\cdot \left(P_{\text{mcu}}+P_{\text{soff}}+P_{\text{toff}}\right)+T_{\text{sample}}\cdot \left(P_{\text{sample}}+P_{\text{sactive}}+P_{\text{toff}}\right)+T_{\text{store}}\cdot \left(P_{\text{soff}}+P_{\text{toff}}+P_{\text{store}}\right)\right)+T_{\text{sleep}}\cdot \left(P_{\text{sleep}}+P_{\text{soff}}+P_{\text{toff}}\right)+N^{-1}\left(T_{\text{nv}}\cdot \left(P_{\text{store}}+P_{\text{soff}}+P_{\text{toff}}\right)+T_{\text{send}}\cdot \left(P_{\text{comm}}+P_{\text{soff}}+P_{\text{send}}\right)\right)$$

where *T*_sleep_, *T*_setup_, *T*_sample_, *T*_send_, *T*_store_, *T*_nv_ are the duration of each respective phase, *P*_sleep_ is the power consumed in sleep mode, *P*_soff_ is the power absorbed from sensors when sleeping, *P*_toff_ is the power consumption of the transceiver when the node is in sleep mode, *P*_mcu_ is the power consumed by the MCU, *P*_sample_ is the power spent for data acquisition, *P*_sactive_ is the power consumed by sensors, *P*_comm_ is the power consumption for filling the transceiver output buffer and, finally, P_send_ is the power for sending data. All of the values for the power consumption and timings are obtained by the values reported in Table 1 measured on real hardware or extracted from the datasheets.

## 6. Results

In this section, we compare the reconstruction performance of CS and the latent variable-based statistical model against the 3ENCULT deployment, considering data coming from the temperature, humidity and light sensors. We want to investigate whether a better reconstruction technique exists among those proposed here and how the sub-sampling parameters affect such recovery quality

We can consider that each node samples the signals for a period of time *T*, called the acquisition period, ideally gathering *N* = *T*/*f_s_* samples at a *f_s_* sampling frequency The compression phase is the same for both frameworks: each node samples the signals of interest gathering a sub-set *M* of the needed samples (*M* = *ρN*), with an under-sampling ratio 0 < ρ < 1. The under-sampling pattern is locally generated by each node using its own ID and the time-stamp as the seed for randomization. In the random sampling pattern, the inter-measurements intervals are always multiples of the minimum sampling period *T_k_* = *T*/*N*.

After the acquisition period *T* = *NT_k_*, the gathered data are sent to the collecting sink through the network. The sampling time *T_k_* in the following simulations is set to 600 s and the results are averaged over 100 trials. Each trial is characterized by a different sampling pattern and a different considered portion of the signal. The reconstruction phase is fairly different and determines the recovery quality of the original signal. For CS, the DCTmatrix is used as the sparsifying matrix, which has already been demonstrated to be a good sparsifying matrix for natural signals [45,46].

In the first simulation, we reconstruct the original signals from a sub-sampled version without exploiting any inter- or intra-correlation among them, just averaging the reconstruction quality over all of the signals, with a signal length of *N* = 512. For the latent variables approach, here, we used the standard CP tensor factorization technique, without the contribution of any correlations in the data. The comparison is carried evaluating the signal-to-noise ratio (SNR), defined as:

(10) $${\text{SNR}}_{\text{dB}}=20\cdot \log_{10}\frac{{\left\|\text{x}\right\|}_{2}}{{\left\|\text{x}-\hat{\text{x}}\right\|}_{2}}$$

where **x** is the original signal and **x̂** is its recovered version. We show the average SNR across all of the network nodes.

**Figure 4.**
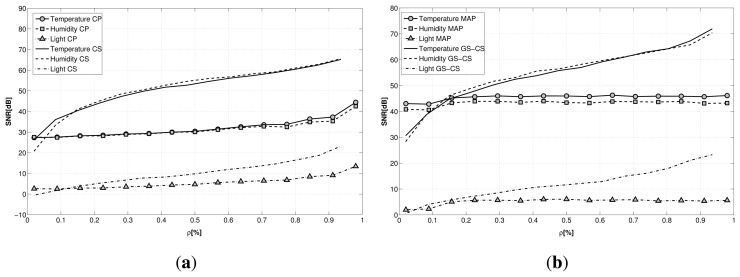
Recovery comparison when reconstructing the original signals from sub-sampled versions (*N* = 512): **(a)** compressive sensing (CS) and latent variable (LV) tensor factorization averaging the reconstruction quality over all of the nodes; **(b)** GC-CS and maximum *a posteriori* (MAP), exploiting the correlations among sensors and nodes.

From the plot, shown in Figure 4a, we can infer how different the performance is for the two techniques: while the reconstruction performance for LV is pretty stable when varying the sub-sampling factor ρ, CS is much more affected by the compression factor. Recovery with CS achieves a better reconstruction almost for every sub-sampling factor with respect to the latent variable-based technique.

Both of the frameworks seem to be greatly affected by the nature of the signal to reconstruct. In particular, from the plot, we can infer how difficult the recovery of light signals is for the two proposed techniques. This is due to the nature of the light signal that is recorded inside the building. While for temperature and humidity, the gathered signals are continuous signals and smoothly affected by human presence, the light signal is highly irregular and highly influenced by the artificial lighting in single rooms. Moreover, some of the nodes are placed in the basement, where the light level is under the noise threshold of the light sensors, providing extremely noisy data.

To evaluate whether it is possible to exploit the correlations existing among sensors and nodes to improve the reconstruction, we performed the recovery against the same dataset using for CS the group sparse optimization (GS-CS), exploiting the joint sparsity of the signals, and for LV the maximum *a posteriori* optimization (LV-MAP) introduced in Equation (8). The results are reported in Figure 4b. While the performance for CS remains almost the same, the LV-MAP method guarantees a significant improvement in reconstruction, resulting in better performance than CS for small values of ρ, especially in relation to humidity and temperature signals.

**Figure 5.**
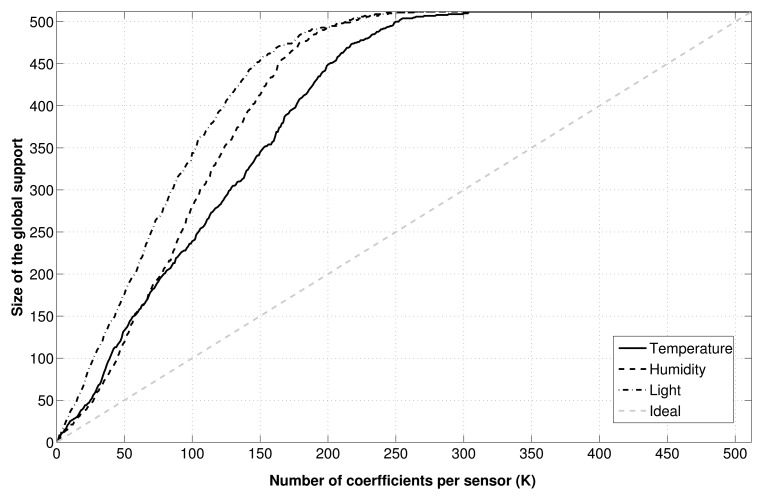
Number of DCTcoefficients necessary to include the *K* largest coefficients for each signal (*N* = 512).

The behavior of CS could be explained by looking at Figure 5. Here, we show how the union over all signals of the *K* best DCT basis vectors per signal has a size definitely greater than *K*. Practically, this means that GS-CS is able to exploit the inter-node correlation only to a small extent, since the shared information among different nodes is limited, and the recovery algorithm is not able to exploit this information to improve the recovery quality.

According to the model in Section 5.2, the simulations are performed with a sampling frequency *f_s_* = 1/600 Hz, and since the length of the data is *N* = 512, this brings a delay in the data delivery towards the data collector of 3.5 days. Thus, the size of the recovered signal spans across 3.5 days. Having high values of *N* means that we have to wait a longer time to proceed with data recovery. Therefore, we want now to investigate how the length of the block of data gathered by the sensors affects the two frameworks and whether a correlation between recovery performance and the *N* parameter exists for GS-CS and LV-MAP.

**Figure 6.**
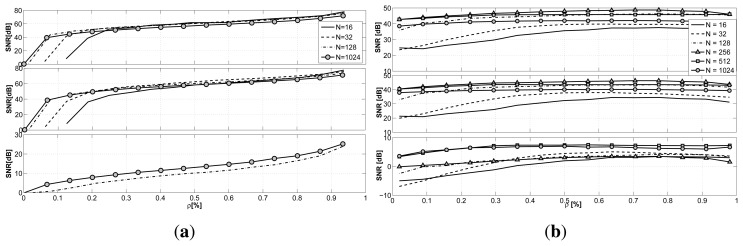
Reconstruction quality varying the sub-sampling factor ρ and using the signal length *N* as the parameter: **(a)** group sparse (GS)-CS and **(b)** LV-MAR From top to bottom: temperature, humidity, light.

In Figure 6a, the results for GS-CS when *N* is changed are presented. From the plot, we can infer how the length of the signal *N* does not greatly affect the reconstruction quality for all of the signals taken into consideration. Rather, we can see how the influence of the parameter *N* (and then, of the delay in the data collection) is only visible for small values of the sub-sampling factor ρ. Different from temperature and humidity, the light signal presents a peculiar behavior showing an increased reconstruction quality with the increase in the number of acquired samples.

The same results for the LV-MAP approach are presented in Figure 6b. The difference in the reconstruction error for the various values of *N* is more evident than in the GS-CS case. With small values of *N*, we registered difficulties in reconstructing the desired signals. The best recovery performance is achieved when considering 256–512 samples at a time, identifying the optimal trade-off between delay and reconstruction accuracy, since larger blocks of data present again a loss of accuracy. For a delay smaller than *N* = 128, the reconstruction of the light signal is not feasible in both cases, since the majority of the samples gathered are zeros, due to the lack of light at night.

**Figure 7.**
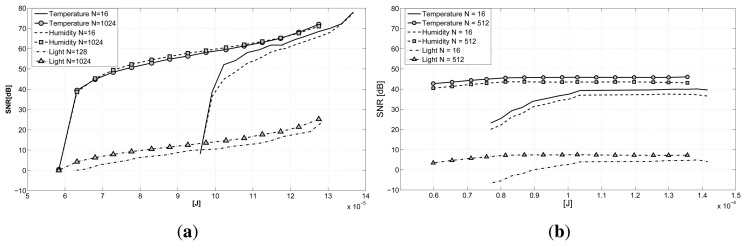
Reconstruction quality *vs.* averaged per cycle energy consumption varying the parameter *N*: **(a)** CS and **(b)** LV.

Having evaluated the recovery performance and the influence of the gathering delay on reconstruction, it is interesting to investigate the power consumption involved with compression according to the power model in Section 5.3. In Figure 7, we report the reconstruction quality against the energy consumption for one acquisition cycle. The plot clearly shows how a trade-off between energy consumption and transmission delay does exist in the GS-CS case (Figure 7a). Higher values of *N*, thus higher delays in transmission, are able to guarantee a better reconstruction quality with definitely less energy than the *N* = 16 case (all of the other cases are not considered in the plot, since they are between these two boundaries). The light signal is a special case, but we can draw the same conclusions as before. The LV-MAP case (Figure 7b) presents a similar behavior, but with a less emphasized increase in energy efficiency corresponding to the increase in data size *N*. When comparing the two graphs, we can observe how the GS-CS case exhibits a slightly higher energy efficiency, allowing a higher reconstruction quality when considering the same energy consumptions as the LV-MAP case. Only for extremely sub-sampled signals are the LV-MAP approach's results better, having a major benefit from the explicit correlation models incorporated in the data reconstruction. In both cases, we are able to obtain a better accuracy (or the same reconstruction quality with less energy) if we are willing to wait for a higher number of gathered samples before proceeding with the reconstruction.

**Figure 8.**
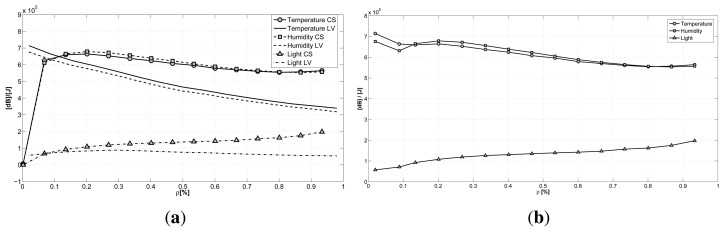
**(a)** Ratio between the recovery quality and energy spent in compression varying the sub-sampling factor ρ for the two approaches; **(b)** the combination of the two approaches.

The same conclusions are shown in Figure 8a, where the ratio between the reconstruction quality and the consumed energy is plotted against the sub-sampling factor ρ. Here, we can see a direct comparison of the two techniques for the case with the best reconstruction performance (*N* = 512). Again, we can see how the GS-CS case has a higher ratio when compared to the LV-MAP case, for almost all of the sub-sampling policies. Only when dealing with a really small amount of sampled data (20%) does the LV-MAP case show a better performance. This result can be a guide for WSN developers, suggesting the adoption of the LV method only when a really aggressive power saving technique is needed. The plots of the two techniques are combined in Figure 8b, where the best ratio between the reconstruction accuracy and the consumed energy is plotted against the sub-sampling factor. Here, we have the combination of the two approaches and show the best achievable results for every sub-sampling factor.

There is no detailed analysis in the literature on conjunct sub-sampled data reconstruction and accurate power consumption estimation for heterogeneous sensor networks. In [28], an SNR of 40 to 68 dB is reported for the reconstruction of temperature signals from compressed data gathered at sub-sampling factors ranging from 0.1 to 0.8, but no power analysis is performed. The hybrid CS aggregation scheme of [18] achieves 20% to 50% energy savings for different aggregation factors, when compared to non-aggregation techniques on a simulated 1255-node grid network. The paper, however, does not report any detail on the power consumption models used, nor on the impacts of the proposed CS aggregation algorithm on the reconstructed data quality. In this work, we presented two energy-efficient approaches, with detailed data reconstruction and power consumption analysis on a heterogeneous WSN, introducing the use of spatio-temporal correlations to improve the performance. Considering the temperature signal, we achieve 40 to 70 dB of SNR for increasing sub-sampling factors, which corresponds to average power consumptions ranging from 63 to 128 μ**J**, with maximum energy savings of 58%. Our results are aligned with the presented literature and introduce detailed multi-signal reconstruction and power analysis based on a real-life WSN deployment, which provide insights into the application of sub-sampling in heterogeneous WSNs and can serve as guidelines and future design specifications.

## 7. Conclusions

In this paper, we presented a wireless sensor network deployment and used it to compare two promising techniques for energy-efficient data gathering and reconstruction. One is based on the well-known compressive sensing framework, while the second is a latent variable-based statistical model. Both approaches try to successfully recover the desired signal from a highly incomplete sub-sampled version, obtained opportunely, skipping samples during the acquisition phase. The two techniques exploit redundancies and correlations present in the gathered data to achieve a better reconstruction accuracy with a smaller number of collected samples and, thus, with a lower energy consumption. We described the hardware and software implementation of the deployed network, introduced an energy model for the sensor nodes and analyzed the gathered data, comparing the two reconstruction techniques. The results showed how the use of the data from the whole network and their correlations improved the recovery performance in both cases, when compared to the standard approaches, where individual nodes and signals are considered. The CS approach usually achieves better reconstruction accuracy, with the exception of cases when really aggressive sub-sampling policies are used. This leads also to the better energy efficiency of the CS method.

## Figures and Tables

**Table 1.** Characteristics of the device taken as a reference in the power model.

***T*_sample_**	**600 (s)**	**Sampling Period**
*T*_setup_	5e–4 (s)	Setup time
*T*_store_(16 bit)	5.25e–5 (s)	Time to store data in NVM
*V*_batt_	3.3 (V)	Battery voltage
*A*_sleep_	1.3e–6 (A)	Sleep current
*A*_mcu_	7.5e–3 (A)	Idle current
*A*_sample_	l.le–3 (A)	ADC current
*A*_store_	7.5e–3 (A)	Current when saving in NVM
*A*_comm_	1e–6 (A)	Current for filling the transceiver buffer
*F*	24 (MHz)	Microcontroller frequency
*A*_send_	3.1e–2(A)	Transmission current
*A*_toff_	10e–6 (A)	Sleep current of the transceiver
*S*_tx_	150 (kbps)	Transmission throughput
*T*_setup_radio_	5e–3 (s)	Transceiver setup time
*B*_pkt_	127 (byte)	Packet size
*B*_header_	10 (byte)	Header size
*A*_sactive,TH_	3e–4 (A)	Temp and Hum.(TH) current consumption
*A*_soff,TH_	1.5e–7 (A)	TH sleep current
*T*_sampl,TH_	2e–5 (s)	TH sampling time
*A*_sactive,AL_	150e–6 (A)	Ambient light (AL) current consumption
*A*_soff,AL_	0.01e–6 (A)	AL sleep current
*T*_sampl,AL_	1e–5 (s)	AL sampling time
